# Leveraging large language models for automated detection of velopharyngeal dysfunction in patients with cleft palate

**DOI:** 10.3389/fdgth.2025.1552746

**Published:** 2025-03-28

**Authors:** Myranda Uselton Shirk, Catherine Dang, Jaewoo Cho, Hanlin Chen, Lily Hofstetter, Jack Bijur, Claiborne Lucas, Andrew James, Ricardo-Torres Guzman, Andrea Hiller, Noah Alter, Amy Stone, Maria Powell, Matthew E. Pontell

**Affiliations:** ^1^ Data Science Institute, Vanderbilt University, Nashville, TN, United States; ^2^ Department of General Surgery, Prisma Health Greenville, Greenville, SC, United States; ^3^ Department of Plastic Surgery, Vanderbilt University Medical Center, Nashville, TN, United States; ^4^ Department of Otolaryngology, Vanderbilt University Medical Center, Nashville, TN, United States; ^5^ Division of Pediatric Plastic Surgery, Monroe Carell Jr. Children’s Hospital, Nashville, TN, United States

**Keywords:** velopharyngeal dysfunction (VPD), hypernasality detection, artificial intelligence (AI), cleft palate, machine learning (ML), speech diagnostics

## Abstract

**Background:**

Hypernasality, a hallmark of velopharyngeal insufficiency (VPI), is a speech disorder with significant psychosocial and functional implications. Conventional diagnostic methods rely heavily on specialized expertise and equipment, posing challenges in resource-limited settings. This study explores the application of OpenAI's Whisper model for automated hypernasality detection, offering a scalable and efficient alternative to traditional approaches.

**Methods:**

The Whisper model was adapted for binary classification by replacing its sequence-to-sequence decoder with a custom classification head. A dataset of 184 audio recordings, including 96 hypernasal (cases) and 88 non-hypernasal samples (controls), was used for training and evaluation. The Whisper model's performance was compared to traditional machine learning approaches, including support vector machines (SVM) and random forest (RF) classifiers.

**Results:**

The Whisper-based model effectively detected hypernasality in speech, achieving a test accuracy of 97% and an F1-score of 0.97. It significantly outperformed SVM and RF classifiers, which achieved accuracies of 88.1% and 85.7%, respectively. Whisper demonstrated robust performance across diverse recording conditions and required minimal training data, showcasing its scalability and efficiency for hypernasality detection.

**Conclusion:**

This study demonstrates the effectiveness of the Whisper-based model for hypernasality detection. By providing a reliable pretest probability, the Whisper model can serve as a triaging mechanism to prioritize patients for further evaluation, reducing diagnostic delays and optimizing resource allocation.

## 1 Introduction

Cleft palate affects approximately 1 in 700 live births worldwide and requires surgical intervention during infancy to prevent adverse feeding, speech, and developmental outcomes (1–3). Despite corrective surgery, up to 30% of patients develop velopharyngeal dysfunction (VPD), a speech disorder marked by hypernasality and reduced intelligibility (4–6). VPD significantly impairs communication and has profound psychosocial consequences (7–9). An accurate diagnosis of VPD relies on a perceptual speech analysis by specialized speech-language pathologists (SLPs), often with adjunctive testing with videonasoendoscopy, nasometry and different types of imaging (10, 11). As such, the diagnosis of VPD is highly dependent on specialized expertise and costly testing equipment. Both factors make VPD care nearly inaccessible in low- and middle-income countries (LMICs). As a result, there is an unknown number of patients who remain undiagnosed and untreated, further perpetuating disparities in care for orofacial cleft patients in LMICs (12–15).

Efforts to increase capacity in the diagnosis and treatment of VPD have harnessed the power of artificial intelligence (AI) and machine learning (ML). These models autonomously conceptualize non-linear relationships in data, making them particularly well-suited for nuanced tasks such as VPD detection. Multiple teams have explored traditional ML approaches using support vector machines (SVMs) and random forest (RF) classifiers, utilizing engineered features like Mel Frequency Cepstral Coefficients (MFCCs) to identify patterns in audio data (16–18). While these methods have demonstrated some effectiveness, their reliance on extensive preprocessing and feature engineering limits their practicality, especially in real-world settings (16–18). Similarly, deep learning models such as convolutional neural networks (CNNs) have shown promise but typically require large, annotated datasets, often amounting to thousands of hours of audio, to achieve clinically meaningful performance (19, 20). Furthermore, many of these models are restricted to analyzing specific phonetic sounds or operate within narrow linguistic contexts, which can hinder their generalizability across heterogenous populations and languages (16–20).

Recent advancements in Large Language Models (LLMs), particularly OpenAI's Whisper model, offer a promising approach to VPD detection by leveraging pre-trained audio processing capabilities (21). Unlike conventional models that require extensive preprocessing and manual feature engineering, Whisper autonomously extracts acoustic data directly from raw audio files, enhancing efficiency and real-world applicability. By utilizing a transformer-based architecture trained on multilingual datasets, Whisper excels at capturing subtle acoustic variations, making it well-suited for detecting hypernasality and other speech irregularities associated with VPD. Its architecture is inherently designed to accommodate diverse linguistic contexts, allowing for seamless integration across varied speech patterns and dialects (21, 22). This versatility is particularly valuable in low- and middle-income countries (LMICs), where linguistic diversity and resource limitations pose significant diagnostic challenges (23). With targeted refinements, Whisper can further enhance existing diagnostic methods, improving accessibility and broadening its clinical utility. Despite this potential, Whisper's utilization in VPD detection remains largely unexplored, presenting an opportunity to advance global healthcare equity through AI-driven speech analysis.

The aim of this study is to leverage Whisper's pre-trained audio processing capabilities to develop a model that can automatically detect the presence of VPD by voice sample alone. We hypothesize that Whisper's key encoded features can be repurposed to identify patterns of VPD within voice samples, with a primary endpoint of model accuracy.

## 2 Methods

This study was approved by the Institutional Review Board at Vanderbilt University Medical Center/Monroe Carell Jr. Children's Hospital (IRB#212135). Audio samples of patients with a diagnosis of VPD, as well as unaffected voice samples, were sources from publicly available online repositories and institutional datasets to ensure a diverse representation of speech patterns. Unaffected audio samples were sourced from the Centers for Disease Control and Prevention and the Eastern Ontario Health Unit (21, 22). VPD voice samples were obtained from multiple publicly available sources (23–29).

All recordings were preprocessed into WAV format and resampled to 16 kHz to ensure compatibility with the Whisper model. To standardize inputs, each recording was processed to fit Whisper's fixed 30 s input window by zero-padding shorter samples and truncating longer ones. Metadata, including recording conditions and file duration, was cataloged for each sample.

Patient-level variables, including age, sex, and severity of hypernasality, were not included in the analysis due to the lack of this information in the publicly accessible datasets.

### 2.1 Study design

This study involved data preprocessing, adapting the multi-lingual Whisper model for binary classification tasks, and comparing its performance against traditional machine learning models. The models evaluated included Whisper-base, Whisper-medium, and Whisper-large-v2, each paired with a custom classification head. Baseline comparisons were conducted using Support Vector Machine (SVM) and Random Forest (RF) classifiers.

### 2.2 Whisper model variants

All three variants—Whisper-base, Whisper-medium, and Whisper-large-v2—share the same transformer-based architecture but differ in parameter size, which influences their computational efficiency and ability to capture complex speech features. Whisper-base, the smallest model, prioritizes speed but has lower precision. Whisper-medium offers a balance between performance and computational demand, while Whisper-large-v2, the most complex variant, has the highest number of parameters and was trained for additional epochs to improve accuracy (24).

### 2.3 MFCC extraction for baseline models

For the baseline models, MFCCs were extracted using the LibROSA library in Python (Python Software Foundation, Wilmington, DE) (25). To ensure consistency across varying recording lengths, the extracted MFCC sequences were mean-aggregated over time, generating a fixed-length feature vector. These processed representations were then used as inputs for the SVM and RF classifiers.

### 2.4 Model architecture and training

**Figure 1:**
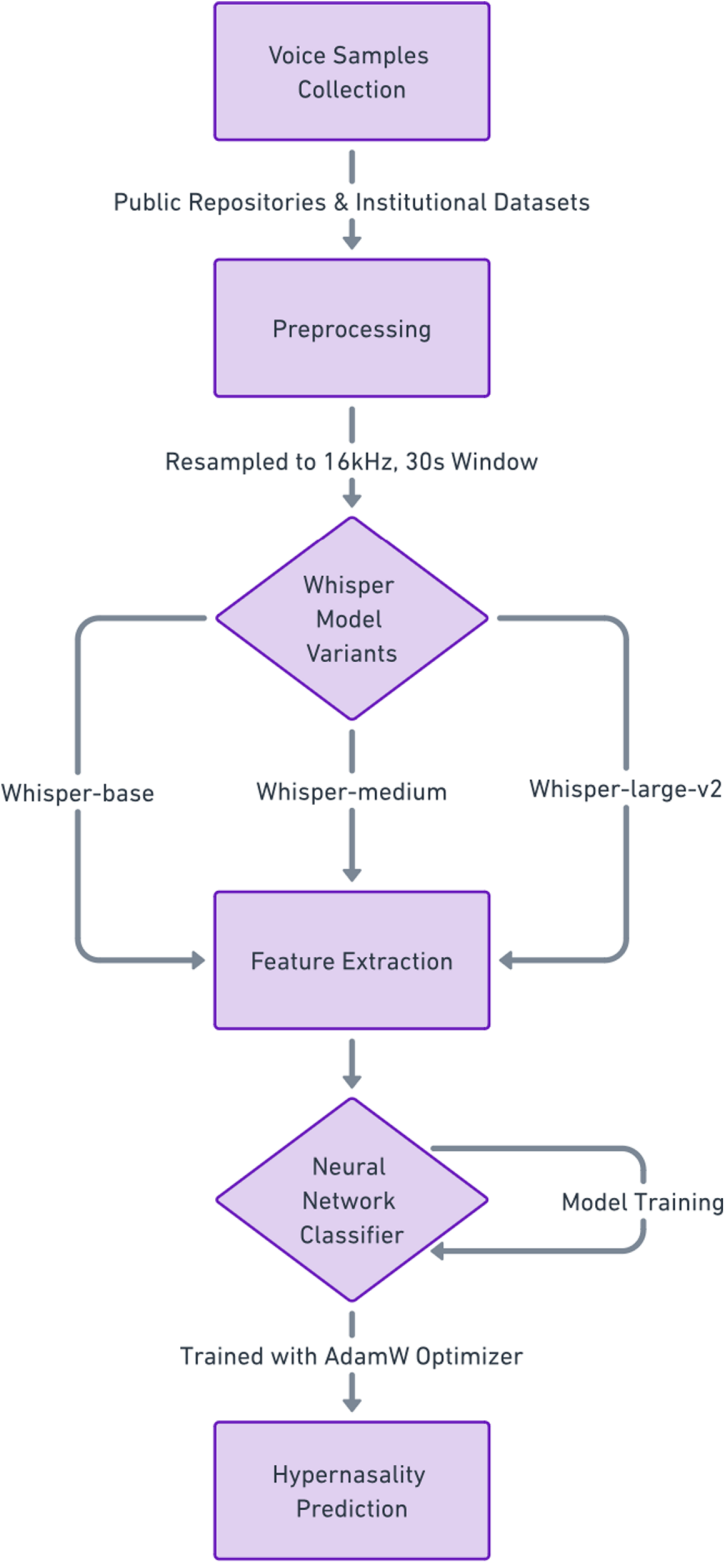
Workflow of the whisper-based model for hypernasality detection.

The Whisper model, originally designed for robust speech-to-text transcription, was adapted for binary classification of VPD. This was achieved by replacing its sequence-to-sequence decoder with a custom classification head. (Figure 1) Each encoder processed the audio data, passing the extracted features to a neural network classifier. The classification head is comprised of five fully connected layers with progressively decreasing output dimensions (4096, 2048, 1024, 512, and 2 nodes), employing Rectified Linear Unit (ReLU) activations between layers. (Table 1) A softmax activation function in the final layer produced probabilistic outputs for classification.

**Table 1:** Neural network architecture for hypernasality detection.

Layer	Operation	Output shape
Linear	Fully connected	4,096
ReLU	Activation	4,096
Linear	Fully connected	2,048
ReLU	Activation	2,048
Linear	Fully connected	1,024
ReLU	Activation	1,024
Linear	Fully connected	512
ReLU	Activation	512
Linear	Fully connected	2
Softmax	Activation	2

To optimize computational efficiency, the pre-trained parameters of the Whisper encoder were frozen, allowing the classification head to focus on learning task-specific features. The model was trained for 10 epochs using the AdamW optimizer, with a learning rate of 0.00002 and weight decay of 0.0005. (Table 2) Cross-entropy loss was used as the objective function, and early stopping with validation monitoring was implemented to prevent overfitting. All training and evaluation were conducted on an NVIDIA DGX A100 GPU.

**Table 2:** Training optimizer and hyperparameter configuration for model training.

Training optimizer	
Optimizer	Hyperparameters
AdamW	Learning Rate: 0.00002
	β1: 0.95,
	β2: 0.95,
	λ: 0.0005

### 2.5 Baseline comparisons

Baseline models, including SVM and RF classifiers, were implemented for comparative analysis. These models utilized MFCCs as input features, requiring extensive feature engineering and preprocessing. Identical data splits were used to benchmark the performance of the Whisper-based model against these traditional approaches.

### 2.6 Performance evaluation

The dataset was randomly divided into training (70%), validation (15%), and test (15%) subsets, maintaining a balanced distribution of VPD and non-VPD samples. Model performance was assessed using metrics such as accuracy, F1-score, and computational efficiency. Validation metrics were monitored during training to identify the best-performing model for final evaluation on the test dataset.

### 2.7 Software and reproducibility

All experiments were implemented using Python, with PyTorch for model training and the Hugging Face library for accessing Whisper encoders. The codebase, including preprocessing scripts and training pipelines, is available in a publicly accessible GitHub repository to ensure reproducibility.

## 3 Results

### 3.1 Dataset characteristics

The dataset included 184 audio samples, with 96 VPD (cases) and 88 non-VPD (controls) recordings. Audio sample durations ranged from 0.44 to 9.35 s. To ensure balanced evaluation across VPD and non-VPD samples, the data was split into training (70%, *n* = 129), validation (15%, *n* = 28), and test (15%, *n* = 27) subsets, maintaining the original 96:88 case-to-control ratio. The final distribution across subsets is shown in Table 3.

**Table 3:** Dataset distribution across training, validation, and test sets.

Dataset split	Total samples	VPD samples	Non-VPD samples
Training (70%)	129	67	62
Validation (15%)	28	14	14
Test (15%)	27	15	12
Total	184	96	88

### 3.2 Whisper-Based model performance

The Whisper-based model demonstrated strong performance across configurations. (Table 4) The Whisper-base configuration, paired with a custom classification head, achieved the highest test accuracy of 97.0% and an F1-score of 0.97. Whisper-medium and Whisper-large-v2 configurations achieved test accuracies of 94.9% and 89.2%, with corresponding F1 scores of 0.95 and 0.89.

**Table 4:** Comparison of hypernasality detection models.

Model performance		
Model	Test accuracy	F-1 score
Whisper-base + Classifier	97.00%	0.97
Whisper-medium + Classifier	94.90%	0.95
Whisper-large-v2 + Classifier	89.20%	0.89
SVM	88.10%	0.86
RF	85.70%	0.88

### 3.3 Baseline model comparisons

Baseline models trained using MFCCs as input features showed lower performance compared to the Whisper-based models. The SVM model achieved a test accuracy of 88.1% and an F1 score of 0.86, while the RF classifier achieved a test accuracy of 85.7% and an F1 score of 0.88. These traditional models required significant preprocessing and manual feature engineering, which increased computational overhead.

## 4 Discussion

This study demonstrates the effectiveness of OpenAI's Whisper model for automated VPD detection, achieving a test accuracy of 97% and an F1-score of 0.97. These results significantly outperform baseline models, including SVM (88.1% accuracy) and RF classifiers (85.7% accuracy), which relied on handcrafted features such as MFCCs. Whisper's ability to capture nuanced speech characteristics directly from raw audio samples, coupled with its holistic processing capabilities, underscores its value in both technical performance and clinical utility. These findings validate the feasibility of leveraging ML technology to bridge gaps in diagnostic care, particularly in underserved and resource-constrained settings.

Conventional methods for hypernasality detection rely heavily on perceptual assessments conducted by SLPs and adjunctive tools such as nasometry, videofluoroscopy, or imaging systems (10, 11). While effective, these methods pose substantial barriers due to significant costs, reliance on specialized equipment, and the need for highly trained personnel. These challenges are particularly pronounced in LMICs, where healthcare infrastructure is limited, and access to qualified professionals is often scarce (13, 15, 30, 31). As a result, many patients in these regions remain undiagnosed and untreated, exacerbating the functional and psychosocial burdens associated with VPD (12–15).

The Whisper-based model provides an innovative solution by offering a high pretest probability of VPD, ensuring efficient triage of patients most likely to benefit from specialized care. By reducing unnecessary referrals and diagnostic procedures, the model minimizes financial and operational waste for healthcare providers and families (32, 33). These benefits are particularly relevant in LMICs, where the cost of consultations, procedures, and follow-up care can prohibit access to care (34–36).

The success of the Whisper-based model lies in its technical architecture. Whisper's pre-trained encoder autonomously extracts high-dimensional acoustic features, such as pitch, tone, and resonance, directly from raw audio data, providing a rich foundation for downstream tasks and eliminating the need for extensive preprocessing (21). Unlike traditional models that process segmented audio, Whisper holistically analyzes entire audio samples, enhancing its clinical applicability. By replacing its sequence-to-sequence decoder with a classification head, Whisper's encoded features can be repurposed for binary classification of VPD detection. This modular design not only minimizes computational demands but also preserves the integrity of the learned representations, enabling efficient and accurate classification of VPD. Importantly, the model demonstrated consistent accuracy across diverse recording conditions, underscoring its resilience to variability in data quality, linguistic diversity, and speaker characteristics, a critical attribute for global healthcare applications in LMICs.

Interestingly, Whisper-base outperformed Whisper-large in hypernasality detection, an unexpected finding given the typical advantage of larger models in speech-related tasks. One likely explanation is overfitting, as Whisper-large's greater parameter count may have captured irrelevant speaker variations, background noise, or linguistic structures rather than the core acoustic features of hypernasality. Additionally, because Whisper was originally designed for speech-to-text transcription, larger models may allocate more resources towards linguistic structure and phoneme recognition, which are not directly relevant to hypernasality classification. In contrast, Whisper-base's streamlined architecture may have retained the essential acoustic features necessary for detecting hypernasality without over-prioritizing language modeling. Furthermore, freezing the encoder may have disproportionately affected Whisper-large, as its deeper architecture depends on layer-wise refinements that could have been disrupted. In comparison, Whisper-base may have been inherently better suited for direct acoustic feature extraction, requiring fewer trainable parameters to adapt effectively to the classification task. These findings underscore the importance of model selection and adaptation in AI-driven speech pathology applications.

The mobile integration of the Whisper-based model represents a logical and impactful next step in improving access to VPD detection and care. With the widespread availability of smartphones, deploying this technology on mobile platforms could democratize diagnostic access. A smartphone-based application could record and analyze speech locally, providing immediate feedback to users without requiring an SLP (37). When combined with cloud computing, the model could support large-scale data analysis, enabling personalized diagnostic insights and more comprehensive population health monitoring (38). This approach would facilitate earlier identification of VPD, expediting referrals for surgical or therapeutic interventions. By reducing diagnostic delays, this technology has the potential to improve long-term psychosocial and developmental outcomes for individuals with VPD (39, 40). Additionally, integrating the Whisper-based model into telemedicine platforms could bridge gaps in care by connecting underserved populations to specialized services (41). This capability empowers community healthcare workers to perform screenings and identify high-risk patients, amplifying the reach of existing healthcare resources.

Despite promising results, this study has several limitations. The dataset was relatively small, consisting of only 184 audio samples, with limited validation and test sets. While the model achieved high accuracy, the small sample size may impact generalizability, particularly across diverse populations, linguistic backgrounds, and recording conditions. Additionally, the study relied exclusively on publicly available data, which may not fully capture the complexity of clinical settings or the variability of patient presentations (42–46). Future research should incorporate proprietary datasets with greater diversity in noise levels, patient demographics, and linguistic contexts. Prospective validation in clinical environments is also needed to assess real-world performance and usability. Additionally, this study did not compare Whisper against a naive neural network, which could provide further insight into the benefits of pre-trained transformer-based models. Exploring this comparison in future research would help contextualize Whisper's performance in hypernasality detection. Lastly, while Whisper's pre-trained encoder demonstrated strong results with English-language samples, additional optimization is necessary to ensure robust performance across non-English languages and dialects, a critical requirement for global scalability.

The strengths of this study lie in its innovative application of OpenAI's Whisper model, which achieves high accuracy and computational efficiency for VPD detection with minimal training data. Additionally, the model's robustness across varying audio conditions makes it highly suitable for real-world deployment. By combining technical innovation with clinical relevance, this study lays the groundwork for deploying intelligent diagnostic tools worldwide, improving care for individuals with cleft-related speech disorders.

## 5 Conclusion

This study demonstrates the feasibility of adapting OpenAI's Whisper model for automated VPD detection by replacing its sequence-to-sequence decoder with a custom classification head. The adapted model achieved a test accuracy of 97% and an F1-score of 0.97, significantly outperforming traditional models such as support vector machines (accuracy of 88.1%) and random forest classifiers (accuracy of 85.7%). These findings lay the groundwork for future AI-driven tools that can expand access to diagnostic and therapeutic care for cleft-related velopharyngeal dysfunction. AI/ML approaches are particularly suited for care delivery in LMICs, where resources are constrained and clinical expertise is often unavailable.

## Data Availability

The raw data supporting the conclusions of this article will be made available by the authors, without undue reservation.
